# Qualitatively exploring the application of the necessity concerns framework to antenatal physical activity

**DOI:** 10.1186/s12884-023-05918-6

**Published:** 2023-08-24

**Authors:** Sinéad Currie, Alison Eadie, Ronan E. O’Carroll

**Affiliations:** Psychology, Faculty of Natural Sciences, University of Stirling, Stirling, Scotland

**Keywords:** Physical activity, Antenatal, Pregnant, Necessity concerns framework, Framework analysis

## Abstract

**Background:**

Adherence to physical activity (PA) recommendations during pregnancy is low. A common reason for low adherence is concern of harm to mother and/or baby. The Necessity-Concerns Framework (NCF), is a well-established framework in medicine adherence, however it has not been used to explore adherence to antenatal PA. This study aims to explore (1) what influences pregnant women’s PA in the context of the NCF; and (2) if the NCF is an appropriate framework to understand antenatal PA engagement.

**Methods:**

Semi-structured interviews were conducted with 18 pregnant women in the UK and Ireland (mean gestation 27 weeks). Interviews explored beliefs, experiences, perceived necessities and concerns about PA. Interviews were transcribed verbatim and analysed using thematic framework analysis.

**Results:**

Five themes were identified as influential to antenatal PA: (1) Perceived benefits and necessity of PA, (2) Concerns regarding antenatal PA, (3) Balancing the necessity and concern, (4) Barriers to antenatal PA, (5) Facilitators of antenatal PA. Women described a number of perceived necessities and concerns regarding antenatal PA. These necessities and concerns were described as being consciously balanced, supporting the NCF. However, a number of additional influences (for example, feelings of nausea and lack of advice and knowledge) seemed to impact antenatal PA engagement before women could consider their perceived necessities and concerns around antenatal PA.

**Conclusions:**

The Necessity Concerns Framework is a useful framework to help explain how and why women engage with antenatal PA, more specifically why women do and do not engage in antenatal PA at different times during their pregnancy. However, there are a number of other interpersonal and intrapersonal influences on antenatal PA (e.g. physical symptoms, motivation and time), suggesting the NCF alone may be too simplistic to understand and influence complex behaviour such as antenatal PA.

## Background

Physical activity (PA) is a modifiable health behaviour. Engaging in regular, moderate intensity PA can bring many benefits to a pregnancy including reduced risk of excessive gestational weight gain, gestational diabetes, gestational hypertension, pre-eclampsia and symptoms of postnatal depression [1]. These benefits are most likely when pregnant women adhere to PA recommendations. Current UK recommendations advocate active pregnancies and encourage healthy pregnant women with no contraindications, to engage in at least 150 min of moderate intensity PA per week, in line with the recommendations for healthy adults [2, 3]. Despite the benefits and recommendations, research from around the world estimates that less than 25% of pregnant women meet PA recommendations [4].

Research demonstrates that many women are aware of the benefits of PA during pregnancy [5] and also express an intention to be physically active when pregnant [6]. However, perceived barriers towards antenatal PA often outweigh the known and perceived benefits [5]. A range of qualitative studies report the most common barrier to participation in antenatal PA being risk perceptions, where women are worried that engagement in PA will put their pregnancy and baby at risk [5–7]. Hence, in order to improve PA engagement and subsequent pregnancy experiences and outcomes, it is vital to fully understand women’s beliefs and develop strategies to translate the existing intention into effective and sustainable action.

One framework which does recognise the impact of concern on behaviour is the Necessity Concerns Framework (NCF) [8]. The NCF was originally applied to medicine adherence and posits that behaviour can be explained by exploring two key beliefs: (1) perceptions of personal need/necessity for the medication or treatment and (2) concerns about the potential adverse consequences relating to the medication or treatment. Meta-analyses have confirmed this effect in a range of populations [9] and studies have shown that interventions based on the NCF can positively influence medication adherence [10]. This literature indicates that addressing concerns and enhancing necessity beliefs are vital for improving treatment adherence. Currently, there is little research extending the application of the NCF to other health behaviours. Given that a major barrier for women engaging in antenatal PA is their concern of risk, the NCF may be a particularly useful framework for understanding antenatal PA engagement as well as developing an individually tailored PA intervention to improve adherence to PA recommendations .

Hence this study aimed to explore (1) what influences pregnant women’s PA in the context of the NCF; and (2) if the NCF is an appropriate framework to understand antenatal PA engagement.

## Methods

### Study design

This exploratory study was carried out in collaboration with the maternity department of a large hospital in Scotland. A qualitative approach using semi-structured interviews was applied to explore participant’s attitudes, beliefs, perceived necessities and/or concerns and practice of antenatal PA. The use of semi-structured interviews enabled flexible data collection, whilst also ensuring that topics relevant to the research question were covered in each interview.

### Ethical approval

The study was approved by the NHS Health Research Authority, South Central - Hampshire B Research Ethics Committee, on the 24th of October 2017 (17/SC/0547). All participants provided written informed consent and were reassured that the information they provided during the interview would be stored securely and would remain confidential and anonymous.

### Eligibility criteria and recruitment

Convenience sampling was used to recruit people currently living in the United Kingdom and Ireland who could speak and understand English. To be eligible to take part, participants needed to be pregnant (at any gestation) and over 16 years old. They did not need to identify as a woman. Participants were initially recruited through contact with their midwives and the study’s research assistant by distribution of study leaflets when they attended routine antenatal appointments at a Scottish NHS hospital. Following this, a second recruitment drive was carried out through online advertisements placed on social media and university platforms. Potential participants who responded to the adverts were then followed up via email containing the participant information sheet, consent form and offered a telephone call to further discuss the study. Written consent was returned electronically or using a pre-paid envelope.

### Participants

Telephone interviews were conducted with eighteen participants. At this stage, a decision was made by the authors that no new information was emerging from the interviews and a decision to cease recruitment was made [11]. Participant characteristics can be seen in Table 1. Participants had a mean age of 32.5 years and a mean gestation of 27 weeks.

All participants received a £10 shopping voucher following the interview.

**Table 1:** Participant Characteristics (N)

	N
**Age (years)**	
25–29	4
30–34	9
35–39	4
40 and over	1
Index of deprivation decile* (1–10, 1 = most deprived)	
1	0
2	0
3	0
4	2
5	5
6	3
7	1
8	2
9	4
10	1
**Employment Status**	
Full- time	11
Part-time	2
Maternity Leave	4
Unemployed	1
**Parity**	
First pregnancy	14
Second pregnancy	4
**Gestation**	
First trimester	0
Second trimester	9
Third trimester	9

*SIMD, 2020 used for participants living in Scotland (N = 8); IMD, 2019 used for participants living in England (N = 6); WIMD, 2019 for participants living in Wales (N = 1). Excluded are those living in Republic of Ireland (N = 1) and postcodes not found (N = 2)

### Data collection

Data were collected by one Research assistant (AE) who was a postgraduate health psychology student at the time. At the beginning of each interview, participants were reminded of their right to withdraw at any point and verbal consent was reiterated. A flexible schedule of open questions was used to guide the interviews, allowing for probing of further information and clarifications where appropriate. Interview questions were developed through consultation of existing literature regarding antenatal PA [5, 7] (Table 2). Interviews lasted around 40 min and were all conducted over the telephone. All interviews were recorded using a digital voice recorder, transcribed verbatim by an independent, professional transcriber and checked for accuracy by the AE and SC.

**Table 2:** Topics covered in interviews

Demographics
Health issues related to pregnancy/preventing physical activity
Physical activity behaviour pre-pregnancy
Physical activity behaviour during current pregnancy
Importance of PA during pregnancy
Pros and cons of PA during pregnancy
Worries regarding antenatal PA
Motivation for PA
Recommendations for PA
Support regarding antenatal PA
Other factors affecting PA

### Data analysis

Data were analysed by the research assistant using a thematic framework method [12] allowing for themes to be developed both from the research questions and from the narrative of research participants. All data were coded and analysed according to the five stages of this method (Table 3). Regular meetings between the authors were held throughout data analysis, facilitating further exploration of participants’ responses, discussion of the generation of themes and subsequent agreement on recurring themes.

**Table 3:** Data analysis process adapted from Pope et al. [13]

Stage	Stage description
Familiarisation	The researcher responsible for data analysis (AE) became familiar with the data by thoroughly reading through transcripts and by listening to audio recordings on numerous occasions.
Identification of a thematic framework	Three transcripts were coded before a meeting between AE and SC to discuss key themes and to construct an initial coding framework. Using this framework, a further five transcripts were coded before a second meeting to discuss, revise and refine the work. This process was repeated until no new themes were generated and the final thematic framework was agreed.
Indexing	The thematic framework was systematically applied to all transcripts using a qualitative data management package (NVivo version 12). Codes were then organised into categories reflecting prominent themes within the data set and those that were valuable for addressing the research question: barriers to PA, benefits of PA, concerns of PA and facilitators of antenatal PA.
Charting	A matrix was created for each theme by abstracting, summarising and charting data for each case and each code within that theme.
Mapping and interpretation	Thematic analysis was carried out on the managed data set by reviewing the matrices and making connections within and between codes and cases. This process was influenced by the original research objectives and by concepts generated inductively from the data.

## Results

Of the 18 participants, seven self-reported that they were meeting or exceeding PA recommendations pre-pregnancy (engaging in at least 150 minutes of moderate intensity PA per week). One reported meeting PA recommendations at the time of the interview (during pregnancy) with the other participants not meeting recommendations.

Compared to their pre-pregnancy PA, all participants reported a reduction and change in their PA in either duration, intensity or type. All participants who reported running prior to pregnancy (N = 8) had stopped either as soon as they found out they were pregnant (N = 3) or by trimester three (N = 5). Walking (N = 12) and swimming (N = 6) were the most common types of PA during pregnancy.

Sixteen of the participants reported sports or exercise activities as a major contributor to their pre-pregnancy PA whereas the other two participants (both of which did not adhere to PA recommendations before or during pregnancy), noted engagement in daily living activities such as dog walking or looking after their child/children.

Five themes were identified in the analysis: (1) Perceived benefits and necessity of PA, (2) Concerns regarding antenatal PA, (3) Balancing the necessity and concern, (4) Barriers to antenatal PA, (5) Facilitators of antenatal PA.

### Perceived benefits and necessity of PA

All participants expressed beliefs about the benefits of undertaking PA, both pre-pregnancy and during pregnancy. The most commonly cited benefit of PA was the impact it had on women’s mental health and wellbeing. PA was described as a way to de-stress and get away from the daily worries of life, something that was reported as being especially pertinent during pregnancy which was described as a particularly worrying time. One participant described the pride she felt for exercising, which motivated her to continue.

*“One of the things that I’ve struggled with in the past is anxiety so when you’re physically active that makes it better, so when I’m out with the dog I don’t think I really never ever think about anything or worry” P1*.

*“I think it’s [physical activity] really good in general mentally for people, to de-stress, unwind and I think certainly during pregnancy when there’s a lot on the go and a lot on your brain it’s quite nice to have that time out to chill out a bit” P11*.

One participant described how remaining physically active during her pregnancy was necessary for her to cope with her changing self-identity as she became accustomed to the change pregnancy would have on her life.

*“If I hadn’t of found a substitute [to the gym] then I think I’d be in a lot of probably, not depressed, but I think I would’ve felt like everything been stripped away from me because I was pregnant, the things I used to do and couldn’t do anymore, so it’s been amazing having the swimming to do instead” P15*.

As well as psychological health and wellbeing, participants believed that PA was beneficial to their physical health. Helping them to maintain a healthy weight during pregnancy and support their body’s ability to cope with the changes associated with pregnancy, as well as reducing their risk of developing gestational diabetes. Participants also described how they believed PA benefitted their baby, promoting and supporting their baby’s development, mood and additionally contributing to a healthy birth weight.

*“When you’re exercising you kinda feel quite proud of yourself and you feel like you’re doing something right for your body and right for the baby” P16*.

Participants reported a number of motivations to exercise during pregnancy, with the most commonly described being the belief that remaining active during pregnancy would lead to a quicker less complex labour, reduced chance of a caesarean section, and faster postnatal recovery. This view tended to be held by women who were exercising during their pregnancy, with participants who were less active or not active not citing this as a motivation to exercise.

### Concerns regarding antenatal PA

Concerns regarding PA varied; most participants interviewed described these concerns as the reason that they reduced or modified their PA throughout pregnancy.

The most frequently described concern was causing harm to the baby: the degree of this concern varied with gestation, participants generally describing the first trimester as the most concerning period, when risk of miscarriage and negatively impacting fetal developed were particularly pertinent. Participants referred to a concern that PA may cause miscarriage or stillbirth throughout gestations, especially if PA included impact, or resulted in them becoming excessively hot, dehydrated, or raising their heart rate significantly.

*“I was worried about, causing the baby, it’s irrational but causing the baby to have a like a mini heart attack because my heart was going too high, cause I heard the traditional advice of don’t go over 140 beats a minute… cause I felt like if my body’s concentrating on keeping me going then, you know, it’s diverting the blood away from the baby” P13*.

Concerns influenced women’s decision making around the type of PA they undertook; for example, some women reported doing less vigorous PA in the first trimester, such as yoga and walking and choosing not to do PA they had previously engaged in such as cycling, weight training; only feeling confident to reintroduce more vigorous PA after the first trimester.

*“Before you have a 20 week scan…the developmental scan… you don’t really feel kicking, you’ve got a little bump but… you don’t really feel like baby is fully viable and for that reason I think you’re just a little bit more cautious, and then after that, once you kinda get into pregnancy, the second half of pregnancy, you are definitely more confident with the exercise that you can do” p16*.

Contrary to this, participants also reported a belief that their baby was more protected from risks associated with PA during the earlier gestations, when their bump was smaller and less likely to get knocked. Participants who held this view reported being less likely to engage in vigorous exercise from the mid-late gestation.

The majority of participants believed that, compared to pre-pregnancy, they should not increase the amount of PA they were doing during their pregnancy, or take up new forms of exercise/sports, with the exception of certain PA’s such as antenatal yoga and Pilates.

Participants were also concerned that PA could harm their own physical health, such as damaging their pelvic floor and abductor muscles or exerting themselves more than they needed to when it was already coping with pregnancy.

For some participants, the concern regarding PA was sufficient to stop them engaging in moderate and vigorous PA completely.

*“Ultimately you are responsible for another life and I just felt that really I didn’t want any risk at all, I just didn’t want to take any risk so I just decided the best thing was to not go [to the gym]”* P17.

### Balancing the necessity and concern

Perceived necessity and concerns regarding antenatal PA were consciously balanced to determine PA behaviour. When necessity outweighed concern, then PA was more likely and when concerns outweighed necessity, PA was less likely.

This balance was continually assessed throughout pregnancy, rather than being fixed throughout, contributing to the varying decisions made regarding the type and intensity of PA performed at different stages of pregnancy.

For some participants, assessing this balance was significant in their decision to stop exercising, describing that whilst they believed that there were many benefits of antenatal PA, the “*cons ultimately overtake them [the pros of PA] because of what they are*” (P2). However, the majority of participants described how the benefits they gained from antenatal PA outweighed any concerns they had and instead of stopping them exercising, their concerns instead prompted them to make changes to the type, duration or intensity of PA, which they believed was important to retain the balance of needs and concern in favour of PA.

*“For me, it’s thinking that the benefits [of PA] to my wellbeing outweigh the risks, of which the risks are smaller because I’m trying to reduce the risks, as I’m going along, and obviously the fact that in general being fit and healthy and continuing with that exercise is good for my body and for the baby as well”* P10.

### Barriers to antenatal PA

A number of barriers were perceived as restricting the amount of PA participants performed. Some of these were physical symptoms associated with pregnancy such as nausea, tiredness, morning sickness, feeling uncomfortable and back pain. These barriers, for some participants, prevented them performing PA, which was often contrary to their intention and motivations. Other barriers were more logistical; multiple participants described that finding maternity clothes suitable for specific sports (cycling, swimming) was both difficult and expensive and that working full time or already having young children made it difficult for them to find the time to prioritise structured PA.

Participants also described how the opinions and beliefs expressed by other people discouraged them from antenatal PA: these reportedly came from personal trainers, family members and also feeling judged when exercising in public whilst visibly pregnant. For example, one participant reported that when she told her personal trainer about her pregnancy, that they seemed worried about working with her, and this promoted her own worry. Similar experiences were described during contacts with healthcare professionals, where the absence of a discussion about antenatal PA or a lack of definitive advice was often interpreted as a concern, therefore acting as a barrier to PA.

*“ When I asked my midwife at my booking in appointment what I could and couldn’t do there was a lot of hesitancy on their part… there was two people… and they were kinda asking each other and they didn’t really have clear cut answers to what I thought were quite straightforward questions like can I keep up my running and things, and they kind of agreed that ‘oh I’m sure if you’ve done it before, they say that’s fine to keep doing it’ but there certainly wasn’t very definitive yes or no … which I think was already quite an anxious time isn’t it, so it wasn’t particularly helpful”* P11.

### Facilitators of antenatal PA

Possessing accurate knowledge regarding antenatal PA was associated with confidence to perform PA and less concern, facilitating the likelihood that women continued to perform antenatal PA throughout pregnancy. Knowledge was often gained through own research, by attending pregnancy specific PA classes and having peer role models who also exercised through pregnancy. Multiple women highlighted how the visibility of other women on social media who exercised during pregnancy encouraged them to exercise.

In addition, participants also described how positive interactions with healthcare professionals encouraged them to perform PA.

*“She [midwife] was definitely encouraging of it [cycling], she didn’t explicitly say “you must keep doing that” or anything like that, but yeah no she seemed quite supportive about it”* P12.

Participants also reported that they felt pressure from society and peers to maintain a healthy weight during pregnancy and this also acted as a facilitator/motivator of antenatal PA.

*“…the pressure of society to not put on loads of weight… but that’s I suppose the other way, which would encourage me to do a bit more [physical activity].* P17.

**Fig. 1:**
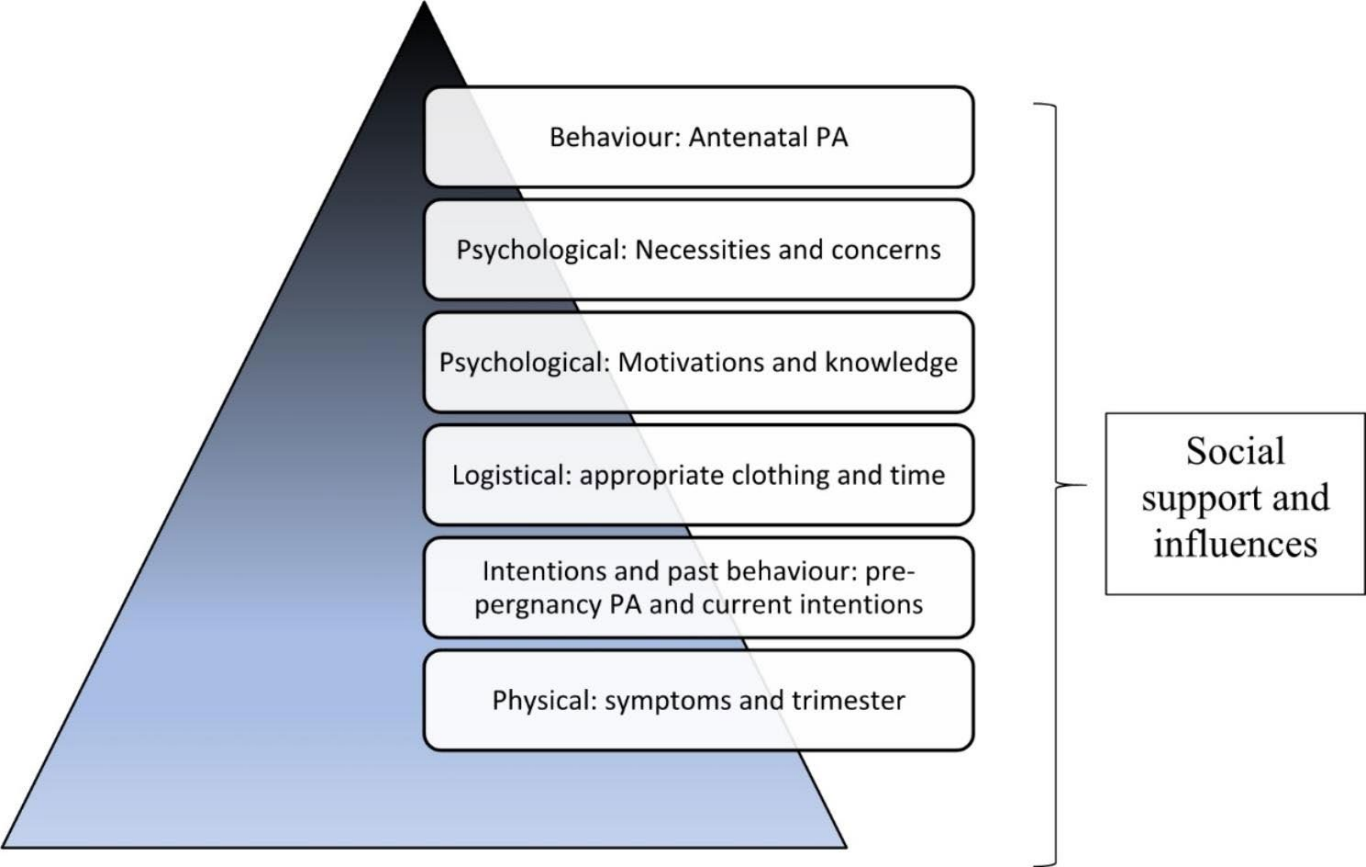
Hierarchy of influences on women’s antenatal physical activity engagement

The data shows that, necessities and concerns seemed to be considered after a range of less cognitive influences were addressed, such as reduced pregnancy symptoms and available time. Figure 1 represents the above data and themes in a hierarchy of influences on womens antenatal PA engagement. Hence, although the NCF is relevant and applicable to antenatal PA, it may fit best when complemented with other theoretical considerations and behaviour change approaches.

## Discussion

Five main themes were identified as influential to antenatal PA: (1) Perceived benefits and necessity of PA, (2) Concerns regarding antenatal PA, (3) Balancing the necessity and concern, (4) Barriers to antenatal PA, (5) Facilitators of antenatal PA. The data show that, although a range of benefits and necessity around being active during pregnancy were cited, concerns and barriers tended to outweigh the necessity. There were a range of other, more predominant factors, which seemed to be immediately influential to PA engagement.

Participants cited a range of perceived benefits and necessity of antenatal PA. External motivation, mainly in the form of benefit to the baby’s health, is a well recognised motivation for antenatal PA [5, 14]. Although improved outcomes for baby can be seen as a motivator to being physically active, this relies on the woman feeling confident that PA will be beneficial rather than potentially damaging. Systematic review evidence corroborates by finding that PA of all intensities tends to decrease gradually from pre-pregnancy to trimester one to trimester three and participation in sports based PA also significantly decreases throughout pregnancy [15]. Data from our study indicated that concerns about risk to baby as a result of PA were evident in all trimesters but were more dominant in trimester one. This is understandable as early pregnancy is associated with the highest level of general pregnancy worry, i.e. there may be something wrong with the baby [16]. Hence, this increased general pregnancy worry alongside a perception of PA potentially causing harm to a baby may explain the cessation and/or reduction in PA from pre-pregnancy.

Participants indicated ever changing thoughts and motivations around PA throughout pregnancy. Indeed, changing cognitive determinants of PA is common during pregnancy. Newham et al. [6] assessed Theory of Planned Behaviour (TPB)[17] constructs in each trimester and found that women had significantly less positive attitudes, perceived behavioural control and intention to be active in their third trimester compared with the first and second trimesters. This dynamic, flexible and individual fluidity of behavioural determinants needs to be recognised when considering promotion of antenatal PA.

Those participants who often felt that the benefits of PA outweighed the concerns, reflected that PA was necessary to protect their own wellbeing, in order to provide the best environment and outcomes for their baby. Research supports this belief by women, as mother’s antenatal stress and anxiety is linked to adverse effects on baby development [18]. In addition, research has indicated that those women who engage in the recommended amounts of PA during and after pregnancy (i.e. 150 min of moderate intensity PA per week) are less likely to experience symptoms of depression and anxiety [19]. Hence ‘self-care’ as a motivation to engagement in antenatal PA is, in fact, beneficial to both mother and baby.

Although many women felt the concerns of antenatal PA were outweighed by the benefits and necessity, logistical and social influences often contributed to the intention-behaviour gap. Influences which were ever changing and out with their control such as pregnancy symptoms including nausea and fatigue, often hindered their perceived ability to engage in PA. There has been extensive research into the intention-behaviour gap [20], which has aimed to determine the key factors that are effective in translating intention into actual behaviour. Planning, maintenance self-efficacy and action control are recognised as key volitional variables for translating intention into behaviour [20]. However, considering the ever changing physical, psychological and social aspects of pregnancy, these volitional variables may be challenging to achieve. For example, confidence in maintaining PA levels (maintenance self-efficacy) may be hard to achieve as a woman’s pregnancy symptoms (e.g. nausea and fatigue) and risk factors (e.g. blood pressure) may change throughout trimesters, thus making things unpredictable and reducing women’s confidence in being able to maintain their PA. This ever changing and potentially wavering confidence may also be influenced by social factors; from our data, the impact of other people was seen as both a barrier and facilitator to PA. Friends, family and healthcare professionals who were not confident at supporting or educating women about appropriate antenatal PA were cited as barriers whereas those who were confident were seen as facilitators. Midwives themselves have recognised their potential influence on women’s PA levels but also express the challenges they face in appropriately supporting antenatal PA. For instance, a qualitative study with midwives by De Vivo and Mills [21] found that many midwives identified that they possessed a lack of training, knowledge and confidence in providing antenatal PA advice. In addition, midwives reflected that PA advice is often viewed as a ‘tick box’ exercise and the advice given to women is dependent on the individual midwives’ own expertise and personal views about the relative importance of antenatal PA in comparison to other factors. The information shared and discussed during antenatal appointments can be extremely influential to a women’s interpretation on the importance of antenatal PA. A large systematic review reported that the most frequent source of health information for pregnant women is health care professionals, followed by family and friends and the internet [22]. Women seeking PA advice from their midwives, who are not confident or trained in provision of such information, may leave both parties unfulfilled and lead to PA not being prioritised.

The findings were linked to the NCF as a number of benefits and necessities of PA were identified, alongside a number of concerns, however it was evident that the determinants of antenatal PA are complex with a range of interpersonal and intrapersonal factors recognised. Specifically, logistical (e.g. time), psychological (e.g. motivation), physical (e.g. pregnancy symptoms) social (e.g. perceived norms), behavioural (e.g. past behaviour) and environmental (e.g. weather) barriers and facilitators often presented prior to the purely intrapersonal considerations of necessity vs. concern. A systematic review synthesizing the attitudes, barriers and enablers to PA in pregnant women [23] has emphasized the range and delicate balance of intrapersonal factors (such as pregnancy symptoms) and interpersonal factors (such as family support) which influence antenatal PA. Our findings corroborate this review as participants expressed a range of both interpersonal and intrapersonal factors as barriers and facilitators, regardless of attitude and intention to be active. In addition, another large systematic review has recognised the limited consideration of interpersonal and environmental factors in theories often applied to influence antenatal PA [24]. In our data, necessities and concerns seemed to be considered after a range of less cognitive influences were addressed, such as reduced pregnancy symptoms and available time (Fig. 1). Hence, although the NCF is relevant and applicable to antenatal PA, it may fit best when complemented with other theoretical considerations and behaviour change approaches. It could be posited that, with pregnancy being a time of constant physical and psychological change, theoretical approaches to antenatal PA must be dynamic and consider the influence of ever changing interpersonal and intrapersonal factors, rather than focus purely on stable, cognitive, intrapersonal factors which may be more relevant in less complex and stable behaviours and populations. More complex models such as the Transtherotical Domains Framework (TDF)[25] may be more appropriate in recognising the wide range of inter-and intra-personal determinants of PA, such an approach has been applied by Flannery et al. [26], who investigated enablers and barriers to PA in pregnant women with overweight or obesity using the TDF and Capability, Opportunity, Motivation, Behaviour (COM-B) model [27].

Our finding could be of benefit to health care professionals who want to support women to be physically active during pregnancies. The range of complex inter- and intra-personal factors will vary for everyone. Hence, using our hierarchy of influences to uncover an individual key influences to antenatal PA may allow women to be best supported to engage in, and benefit from PA.

### Strengths and Limitations

The results of this qualitative study must be considered in the context of its strengths and limitations. Data were collected from a range of pregnant women in different trimesters. Hence the data are current reflections, thoughts and experiences rather than retrospective which can be skewed with hindsight. However, it should be noted that there were no participants in their first trimester. This is a common limitation in research as current NHS care in Scotland, including the routine, first dating scan does not occur until roughly 11–14 week gestation, at the end of trimester one [28]. Women were recruited through NHS hospitals and social media which increased the potential diversity of participants.

This study took a unique approach by exploring the application of a theoretical framework through collection of qualitative data. Participants were given the opportunity to provide a full, lived experience of their antenatal PA behaviours and determinants which the researchers then analysed in the context of a theoretical framework. This allows for the model’s relevance to this population and behaviour to be truly applied by exploring all of the potential determinants.

Although recruitment methods were diverse, 78% of the participants were 30 years old or over. This may mean that experiences of younger pregnant women were missed. However, population data from Scotland indicates that in 2018, the average age of mothers was 30.6 years [29]. In addition to age, it should be considered that most of the participants were pregnant with their first child. Previous research has indicated that PA amounts and types differ between first time mothers and mothers with other children [30], possibly due to logistical issues such as childcare and time for recreational PA. Hence our findings may be more applicable to first time mothers.

## Conclusions

This study is the first step in evaluating application of the NCF to an alternative population and behaviour that it was developed to address. The data is qualitative so we cannot definitively say how predictive the framework is in this population and behaviour. However, it does offer a clearer picture as to how it may be applied. In order to explore the predictive validity of the NCF, and proposed conclusions from this data, a large scale quantitative study should be conducted.

Indeed, further work is needed to develop a framework detailing the key psychological, social, biological and behavioural determinants of antenatal PA. This will involve quantitative studies and extensive literature reviews of all studies exploring antenatal PA and application of theory. It may be that a hybrid of theories and experiences help us to understand the balance between intrapersonal determinants such as necessity vs. concerns, and interpersonal factors.

To conclude, the NCF is relevant in helping to understand key determinants of antenatal PA. However, the NCF alone is not enough to fully understand generation and translation of intention into action for antenatal PA. A range of other influences were discussed by women, both intra- and inter-personal (physical and logistical) and these seemed to be more immediate than cognitive or psychological influences such as necessity and concern. Further development of a hybrid of theories to explain antenatal PA is required; however this paper is one of the first steps. Our findings are essential to intervention development as any future intervention to help increase antenatal PA engagement should address the range of intra- and inter-personal influences on PA as well as key psychological influences such as necessities and concerns.

## Data Availability

The anonymized data that supports the findings of this study are available from the corresponding author upon reasonable request.

## List of abbreviations

COM-B: Capability Opportunity Motivation Behaviour model
IMD: Index of Multiple Deprivation
N: Number
NCF: Necessity Concerns Framework
P: Participant
PA: Physical Activity
SIMD: Scottish Index of Multiple Deprivation
WIMD: Welsh Index of Multiple Deprivation
